# Influence of the Viscera of the Human Body upon the Dental Organs

**Published:** 1865-10

**Authors:** Henry S. Chase

**Affiliations:** Iowa City, Iowa


DENTAL REGISTER OF THE WEST.

VOL. XIX.] OCTOBER, 1865. [No. 10.

### Original Essays and Communications.

### "INFLUENCE OF THE VISCERA OF THE HUMAN BODY UPON THE DENTAL ORGANS."

At the last meeting of the AMERICAN DENTAL ASSOCIATION, held at Chicago, Dr. Chesebrough read an Essay on the above subject, which was remarkably able and instructive. The paper is an honor to the Author and the Profession. There is no danger, at present, of too much being said upon Dental Physiology and Therapeutics. We, as a Profession, have, to a great degree, ignored the knowledge and value of this department of study, while we have been busily engaged in improving the mechanical department of Dental *Surgery.*

True, we have arrived at an enviable stand-point in this respect. And *how* has it been done? Let me say, that it is mainly owing to our advancement in Physiology and Dental Medicine in the last few years; which too many, I am sorry to say, seem to undervalue, and grudge the time spent in their discussion. But, just so far as we advance in Dental SCIENCE, just so far shall we advance in Dental ART.

SCIENCE is the STAR which guides the wise men of ART to the cradle of PERFECTION!

*Operative* Dental Surgery will always *claim* and will always *have* its full share of attention—for the greater number of minds are in better adaptation with *it*, than with the facts and theories upon which it is based.

But to return to the subject upon which I wish to make a few remarks. Dr. Chesebrough brought forward some valuable statistics, in his Essay, which proved a much greater tendency to decay in the teeth of women than of men.

I accept the statistics as facts, and believe them to be true. But it seems to me that the Author has gone far out of the way for a cause to account for these facts. He says that "Pregnancy is a natural cause for decay of teeth in women!" I am astonished at such an assertion, and enter my protest against the assumption.

On the contrary I assert that Pregnancy is not, *necessarily*, a cause of decayed teeth.

That Pregnant women suffer more from dental caries than unmarried women, I believe. But, let us carefully examine into the cause of this, and we shall see that Pregnancy is *not* necessarily the destroyer of the dental organs.

I believe that God does not willingly afflict his children. I believe that a natural process, or the exercise of a function of one organ of the body will not, either immediately or remotely, cause the derangement or destruction of another organ of the body.

The latter proposition must be admitted, by all Physiologists, as a Physiological truth.

Well, then, let us look at the discrepancy between it, and the admitted fact that child-bearing women suffer more from decayed dental structures than men.

During Pregnancy women often have perverted appetites, which indulged, lead them into the use of indigestible, innutritive, and injurious articles of diet. Some have a penchant for slate pencils, lemons, pickles, &c., &c. Indigestion, and acidulous saliva follow, and a rapid decay of the teeth follows unless retarded by perfect cleanliness and use of antacids. A craving for slate pencils and such articles, should be met by the exhibition of carbonate of lime, (common chalk) and phosphate of lime, finely ground, and made into small sticks of confection.

Females of the brute creation during pregnancy and lactation indulge a natural appetite, when at liberty to do so, by eating earth, bones, mortar, &c., &c.

Why do these brutes do this?

Evidently to supply the blood with lime salts, which the growth of the *young* either in utero, or born requires.

And this brings us to the *main* cause, the *grand* cause, of dental decay in child-bearing women. *It is the drain upon the mother's blood of lime-salts for new growth of osteogenous and dental tissues.*

I have said that a normal process in one organ should never cause the destruction of another organ! I reiterate the fact.

The drain upon the mother's blood is normal. Obedience to the instincts and laws of nature would supply the blood with abundant osteogenous and dental material.

In *dis*obedience lies the whole trouble.

To *perfect* nutrition in the growing *young*, the mother's health must be *good.* Let her obey the laws of health which are at this day so well known among intelligent persons. In addition to *her* good health, she must not deprive her blood of those elements which the bones and teeth demand, not only in her own body, but in that of her child. The same rule holds equally good, during gestation and lactation.

But how *does* she *deprive* her blood of the elements required?

I answer, by partaking of food which has been *robbed* of those elements.

All food made of the *fine flour* of the cereals has been robbed of them. And does not starch and fine flour constitute the greater part of food used by women?

BREAD, CAKE, CORN STARCH, &c., are samples.

Many women live almost wholly upon bread and butter, and sweet cake.

Unbolted meal of the cereals should be used.

Leguminous articles. Beef, mutton, chickens, game, vegetables. Ripe fruits, natural or preserved. A very moderate amount of tea, coffee and malt liquors may be allowed. Exercise, fresh air, bathing, and SUNSHINE. Does any one say *fudge* at the last word *sunshine?*

Then I am bold to say that the remark proceeds from ignorance.

But I must close, and I hope that I may have thrown some light upon the topic under discussion.

I should have said something upon this subject after Dr. Chesebrough's Essay was read at the American Dental Association, but no opportunity was offered, and therefore I hope he will excuse me for doing so at this time.

HENRY S. CHASE, M. D., D. D. S.

IOWA CITY, IOWA.

